# Supplementary material for: Towards conversational diagnostic artificial intelligence
Source: Nature. 2025 Apr 9;642(8067):442–50. doi: 10.1038/s41586-025-08866-7 (PMC12158756; doi:10.1038/s41586-025-08866-7)
Supplement: Supplementary file 1 — This file contains Supplementary Figs. 1–3 and Tables 1–14. [file 41586_2025_8866_MOESM1_ESM.pdf]

nature portfolio

https://doi.org/10.1038/s41586-025-08866-7

Supplementary information

Towards conversational diagnostic artificial intelligence

In the format provided by the authors and unedited

Supplementary Information

SI.1 Objective structured clinical examination

Objective Structured Clinical Examination (OSCE) is a practical assessment format used in healthcare to assess clinical skills and competencies in a standardized and objective fashion [4, 5, 71]. It differs from traditional written or oral exams that focus primarily on theoretical knowledge and instead aims to provide an environment in which the skills of real-world clinical practice might be assessed.

The OSCE is typically divided into multiple stations (often 8-12), each simulating a real-life clinical scenario enacted by standardized patient actors trained to portray specific symptoms or conditions based on pre-defined scenario descriptions. At each station, students are given specific tasks to perform, such as taking a clinical history, or making a diagnosis. Each station has a set time limit, ensuring fairness and efficient assessment. Trained examiners observe students' performance at each station using a pre-defined checklist or marking scheme. They assess clinical skills like communication, history-taking, physical examination techniques, clinical reasoning, and decision-making.

We designed and conducted a blinded remote Objective Structured Clinical Examination (OSCE) with validated simulated patient actors interacting with AMIE or Primary Care Physicians (PCPs) via a text interface. Our human evaluation aimed at assessing both patient-centered and professional-centered aspects of clinical diagnostic dialogue. We derived 58 axes from the consensus for best practices for patient-centered communication (PCCBP) in medical interviews [19], criteria examined for history-taking skills by the Royal College of Physicians in the UK as part of their Practical Assessment of Clinical Examination Skills (PACES) [20], and criteria proposed by the UK General Medical Council Patient Questionnaire (GMCPQ) for doctors seeking patient feedback as part of professional re-validation.

In particular, PCCBP synthesizes prior literature to derive six consensus categories of function for the medical interview, representing best practice for patient-centered communication: fostering the relationship, gathering information, providing information, decision-making, enabling disease and treatment-related behavior, and responding to emotions. We converted each of these functions into a five-point Likert scale for evaluators to use in assessment. To help calibrate the judgment of evaluators applying Likert scores to each function, we shared the published behaviors given as indicators for how each function might be achieved. For example, in order to foster a relationship with the patient, the authors described that a physician can use appropriate language, show interest in the patient as a person, encourage patient participation, discuss mutual roles and responsibilities, express caring and commitment, engage in partnership building, appear open and honest, and build rapport and connection. We undertook a similar approach to converting PACES items into five-point Likert scales and deriving instructions for evaluators grounded in marksheets published by the Royal College of Physicians which provided evidence of each attribute. Within PACES and PCCBP, all categories were suited for evaluation from a professional-centered perspective, and only a subset of categories also lent themselves for evaluation from a patient-centered perspective. For example, the single category suitable for a patient-centered evaluation in PCCBP was relationship fostering, which allowed us to delve more deeply into this aspect from a patient perspective. We decided to collect more fine-grained annotations from patient actors regarding each of the individual criteria for relationship fostering (e.g., whether the physician used appropriate language, showed interest in the patient as a person, and so forth) to permit a deeper analysis, rather than applying a single five-point Likert scale. For GMCPQ, we implemented the published questionnaire format as is without further adaptation.

Inspired by prior approaches to evaluating LLM generations in clinical settings which noted that both appropriate and inappropriate generations can co-occur and that confabulation or hallucinations may be a risk specific to generative AI systems [12], we additionally evaluated the incidence of both appropriate and inappropriate investigation, management and follow-up plans; alongside looking for evidence of confabulation. As in our prior work, our overall framework items' form and wording were refined by undertaking pilot trials of the assessment and recording feedback from evaluating clinicians. Instructions for the clinicians were written including indicative examples of rating each item for a sample dialogue, and iterated until we observed convergence of the rating approaches by clinicians in the US, India and UK; to indicate our instructions for

evaluation were usable. In our study, we conducted a rating of the physician perspective for all dialogues by a set of three independent physicians (Table SI.1). For robustness, results were reported based on median rating score (or majority vote for binary ratings) among the three independent raters. Additionally, we provided evidence of inter-rater reliability for our framework in Section SI.7.

We undertook a pragmatic approach to derive this usable framework and note that further development, extension and validation would be possible for a measurement instrument, including the application of instrument-construction approaches used elsewhere in social sciences [94].

Table SI.1 | OSCE study summary. Number of scenario packs, patient actors, simulated patients, and primary care physicians (PCPs) in each of the three locations (Canada, India, and the UK) in the remote OSCE study. 20 board-certified PCPs participated in the study as OSCE agents in comparison with AMIE, 10 each from India and Canada. 20 trained patient actors were involved, with 10 each from India and Canada. Indian patient actors played the roles in both India and UK scenario packs. Canadian patient actors participated in scenario packs for both Canada and the UK. This process resulted in 159 distinct simulated patients.

Location	# of Scenario Packs	# of Simulated Patients	# of Patient Actors	# of PCPs
Canada	70	77	10	10
India	75	82	10	10
UK	14	0	0	0
**Total**	**159**	**159**	**20**	**20**

SI.2 Contextualizing our evaluation framework

In contrast to prior works, we anchored our evaluation in criteria already established to be relevant for assessing physicians' communication skills and history-taking quality. We performed more extensive and diverse human evaluation than prior studies of AI systems, with ratings from both clinicians and simulated patients perspective. Our raters and scenarios were sourced from multiple geographic locations, including North America, India and the UK. Our pilot evaluation rubric is, to our knowledge, the first to evaluate LLMs' history-taking and communication skills using axes that are also measured in the real world for physicians themselves, increasing the clinical relevance of our research. Our evaluation framework is considerably more granular and specific than prior works on AI-generated clinical dialogue, which have not considered patient-centred communication best practice or clinically-relevant axes of consultation quality [56, 88, 90–93].

We note that the lay participant perspective was represented in our study by patient actors (not real patients) and it is therefore unclear how accurately their perspectives would represent those of real patients in an actual clinical scenario (since the physical and mental lived experience of real disease symptoms and signs might alter a patients' perspective of the encounter, in ways not replicable by a patient actor). However, as the raters were lay participants who themselves did partake in conversations with the physician (or AMIE), their subjective experience remains valuable to record and analyze, even if the extent to which it is a proxy for a real patient perspective cannot be assured. Furthermore, this possible limitation applies most saliently to evaluation criteria related to changes in the patient's state of mind (e.g., GMCPQ Making Patient Feel At Ease) and less so to criteria regarding the doctor's behavior (e.g., GCMPQ Being Polite), for which it could be expected that lived disease experience may have less impact.

However, our pilot framework is not definitive and can be further improved in future research. History-taking itself is contextual and what determines a "good history" is dependent on the specific clinical situation, patient and physician attributes, cultural characteristics, and many other factors. Despite variation in models for clinical history-taking [95–98], studies have shown that good clinical interviews are associated with not only problem detection and diagnostic accuracy, but also quadruple aims for care delivery [99, 100] ranging from patient and physician satisfaction, resilience to stress and illness, and health outcomes or cost. Future studies on the quality of LLM history-taking might therefore utilise prospective measures of these outcomes in real-world settings (for example reductions in patient complaints [101], or improvements in cost and care effectiveness, patient and provider satisfaction), though evaluations as such may be challenging or impractical to compare to standard practice in the same individual patient, and randomisation of different approaches

may also be challenging in real-world settings.

Our chosen axes of evaluation were not exhaustive and their interpretation was often subjective in nature. Although we conducted evaluations from both clinician and lay-perspectives, generating scenario-packs in three countries with assessors in both North America and India, the pool of clinicians and lay-people assessing the models could be expanded further to improve generalization of our insights. Our experiments could also undergo more extensive replication to explore other aspects such as inter-observer and inter-participant variability, including future work with an intentionally further diversified pool of human raters (clinicians and lay users). Participatory design in the development of model evaluation tools with a representative pool of patients, as well as clinical and health equity domain experts, could also be valuable.

Although our scenarios comprised many different clinical conditions and specialties, our experiments were not necessarily representative of the decades of clinical practice accumulated by even a single doctor (who on average may perform tens of thousands of consultations in a career [102]). The range of conditions possible to examine in medicine is vast as is the variation in presentation of individual diseases. Our experiments were not designed to examine multi-morbidity and co-incident pathology, longitudinal case presentation or the consideration of sequential information from clinical investigations. We excluded entirely some clinical settings or specialties such as psychiatry, pediatrics, intensive care, and inpatient case management scenarios. Further research would be needed to understand the applicability of our findings in many settings such as these, where the requirements for high-quality history-taking might differ [36, 103]. The OSCE framework is commonly used in the assessment of clinicians' skills. It encompasses a significant range of methodologies including real or simulated patients, interaction with physical artefacts or clinical materials, applications to a variety of medical specialties, tasks or settings; and both remote or in-person assessments. Although the OSCE approach is popular, there are significant limitations to its validity [104]. We utilised a remote text-based assessment, replicating known issues with the paradigm of "virtual OSCE" such as the inability to incorporate non-verbal symptoms, signs and communication features. Additionally, this format could introduce unfamiliar constraints to the communication of PCP participants [72].

The tone, content, and nature of the OSCE dialogues in our study are likely not to be representative of real-world patient populations. For example, patient actors may have described their symptoms with greater structure, depth or clinical detail than could be routinely expected in many consultations, or had greater comprehension of clinical context than would be ordinarily expected. Furthermore, although evaluation was blinded, the style of responses from AMIE was notably different to that by PCPs which limits the practical extent of blinding in study design.

SI.3 Auto-evaluation

In addition to human evaluations, we implemented model-based auto-evaluation methods as economical and consistent alternatives to specialist assessments. These techniques were employed to evaluate both dialogue quality and diagnostic accuracy of the OSCE agent.

For the auto-evaluation of differential diagnoses, we leveraged another LLM, Med-PaLM 2 [13] as a surrogate for a specialist rater to grade the predicted diagnoses against the ground truth diagnoses. Our auto-evaluation on DDx accuracy showed a similar trend for AMIE and OSCE agents compared to the specialist ratings. To establish the validity of our auto-evaluation methods for assessing dialogue quality, we initially focused on a subset of four evaluation axes from the PACES rubric (Table ED.1) that were assessed by both the patient actors and the specialist physicians. The auto-evaluation, which uses a self-CoT strategy with AMIE to rate dialogues, was in good alignment with human raters and comparable to the inter-specialist agreement on these criteria. Overall, auto-evaluation trends aligned with human ratings for both dialogue quality and diagnostic accuracy.

In summary, we conducted auto-evaluation analyses for the following purposes:

- To compare the DDx accuracy between simulated patients performed in Canada and India and determine if there is systematic differences between the two locations (Figure ED.4);
- To compare the performance of the DDx accuracy derived from AMIE or PCP consultations (Figure ED.5**a,b**);

- To isolate the effects of information acquisition and information interpretation by analyzing the DDx accuracy of AMIE when provided the PCP consultation instead of its own (Figure ED.5**c, d**);
- To evaluate the efficiency of information acquisition between AMIE and PCPs by analyzing the DDx accuracy as the number of conversation turns increases (Figure ED.6);
- To evaluate the benefit of inner-loop self-play on dialogue quality before and after critic feedback (Figure SI.1).

SI.3.1 Auto-evaluation of DDx

Our auto-evaluation procedure for DDx top-k accuracy is as follows: for each DDx in the DDx list we used Med-PaLM 2 to determine whether the ground truth diagnosis appears within the top-k positions of the differential diagnosis list. Given a prediction and label, the auto-evaluator computes whether they match by prompting Med-PaLM 2 with the following question: "Is our predicted diagnosis correct (Y/N)? It is okay if the predicted diagnosis is more specific/detailed. Predicted diagnosis: prediction, True diagnosis: label Answer [Y/N]:".

Note that both AMIE and Med-PaLM 2 share PaLM 2 as their base model. However, AMIE was fine-tuned on medical dialogue data and optimized for diagnostic conversations, Med-PaLM 2 was designed for the purpose of single-turn medical question answering, and not fine-tuned on medical dialogue data. As such, we considered Med-PaLM 2 sufficiently distinct from AMIE and, as a medical question answering engine, suited for the task of determining whether a predicted diagnosis (e.g., “COPD”) constituted a semantic match given a ground truth diagnosis (e.g., “Chronic Obstructive Pulmonary Disease”).

SI.3.2 Auto-evaluation of conversation ratings

We leveraged the model-based self-CoT auto-evaluation strategy to rate conversations on four evaluation axes from the PACES rubric, and validated that these auto-evaluation ratings were accurate and well aligned with the specialist ratings (Figure SI.1**a, b**). Furthermore, to demonstrate that the inner self-play loop improved simulated dialogue quality, we applied the auto-evaluation method to the simulated dialogues generated before and after the self-play procedure. Results in Figure SI.1**c** revealed that the simulated dialogues after self-play were preferred more often than the baseline dialogues without self-critique. We initially focused on a subset of four clinical axes from the PACES criteria (see Table ED.1), however, this procedure can be easily extended to other ratings.

Self-CoT procedure for auto-evaluation of clinical criteria. The auto-evaluation procedure we employed was a two-step process in which we prompted AMIE itself to rate dialogues on the chosen subset of the PACES criteria (see Table SI.2).

1. First, we prompted AMIE to summarize good and bad aspects of several dialogues and provide an explanation of the provided human rating between 1 and 5.
2. Next, we used these self-generated explanations alongside their respective dialogues as examples in a 5-shot prompt to evaluate and rate a new dialogue. This few-shot prompt included one example for each point on the 5-point rating scale.

In both prompts, we included the rating scale and expert-derived examples of good or bad behaviour for a particular criterion, matching those shown in Table ED.1. We referred to this prompting method as self-CoT (Chain-of-Thoughts) [105] as the plausible reasoning for the human ratings are derived from the model itself.

Rank-order agreement. We evaluated our auto-evaluation method by quantifying its agreement with the specialist rankings of the OSCE dialogues. We limited our analysis to the 159 dialogue pairs in the study. Thus, each pair consisted of a AMIE conversation and a PCP conversation with the same patient actor, and rated by the same three specialists. We treated the median of the three specialist rating as the ground truth rating. For a pair of two dialogues, the three possibilities were: the first one was rated better than the second one, they were equally rated, or the first one was rated worse than the second one. We defined the rank-order agreement as the proportion of dialogue pairs for which the median specialist ranking was preserved by the auto-evaluation ratings. For example, we counted it as correct when our auto-evaluation

rated AMIE's dialogue as better than the PCP's dialogue if the specialists also rated AMIE's dialogue as better, regardless of the exact scores each method assigned.

Auto-evaluation prompting strategies. Using the rank-order agreement metric, we ablated the effect of the two-step prompting and compared it to other methods such as the five-shot prompting (i.e., dropping step 1), shuffled five-shot self-CoT prompting where the order of support examples was randomised each time, and 0-shot prompting using only the rating scale explanation itself (see Figure SI.1**a**). The two-step self-CoT process outperformed the 0-shot and 5-shot methods, and shuffling the examples in the self-CoT prompt made no difference on average.

Benchmarking auto-evaluation. While auto-evaluation was better than random guessing at aligning with specialist preferences, it was unclear if the resulting performance was sufficient. To test this, we computed the average rank-order agreement of the individual specialists as a measure of inter-rater reliability (see Figure SI.1**b**). We observed that auto-evaluation was about as accurate as specialists in predicting the median specialist rankings, suggesting that it is useful to leverage auto-evaluation for these criteria.

Evaluating self-play dialogues. We applied our auto-evaluation procedure to 1,142 dialogues (derived from common conditions) before and after being refined through the self-play critique. We demonstrated that, on average, the refined dialogues after an iteration of critique/revision were rated higher than the original baseline dialogues across all criteria SI.1**c**.

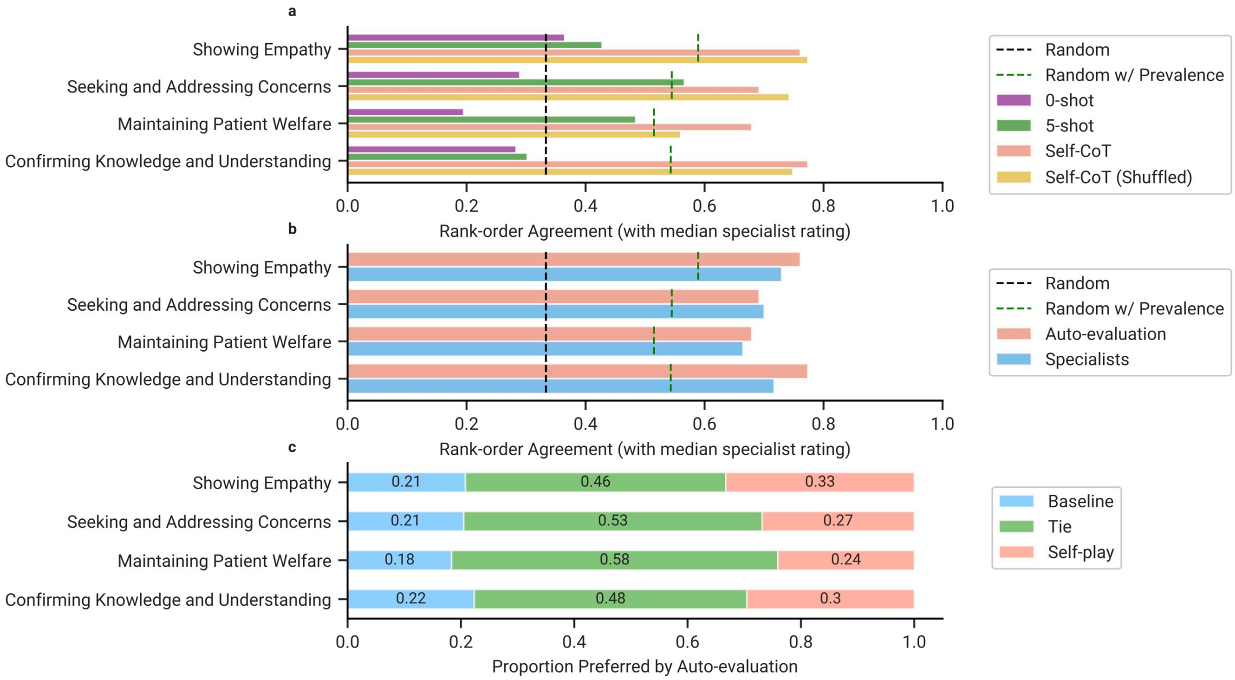

Figure SI.1 | Auto-evaluation of PACES criteria. a, Rank-order agreement (N=159 scenarios) of various auto-eval prompting strategies with median specialist ratings. Randomly guessing, as well as randomly guessing proportional to the prevalence of each option (AMIE, tie, PCP), is indicated with dashed lines. **b,** Rank-order agreement (N=159 scenarios) of the chosen strategy (Self-CoT) with median specialist rating compared to average rank-order agreement of the three specialists with their median ratings. **c,** Preference of self-CoT auto-evaluation between the simulated dialogues (N=1142) before and after one round of self-critique. The self-play dialogues after one round of critique are preferred by auto-evaluation more often than the baseline dialogue generated without revision.

Task	Prompt
Explanation Generation	I have a doctor-patient dialogue and the corresponding rating that quantifies its quality according to the following criterion: <**criterion**> (e.g., maintaining patient welfare). The rating of the dialogue is on a scale of 1 to 5 where:<br>5: <**definition**> e.g., "Treats patient respectfully, and ensures comfort, safety and dignity"<br>1: <**definition**> e.g., "Causes patient physical or emotional discomfort AND jeopardises patient safety"<br><br>First, describe which parts of the dialogue are good with respect to the criterion. Then, describe which parts are bad with respect to the criterion. Lastly, summarise the above to explain the provided rating, using the following format:<br>Good: ...<br>Bad: ...<br>Summary: ...<br><br>DIALOGUE: <**dialogue**><br>Rating: <**human rating**><br>EVALUATION:<br><br>**Example output (for a dialogue with rating 4 on 'maintaining patient welfare'):**<br>Good: The doctor took the patient's concerns seriously and acted quickly to address the situation. They asked pertinent questions to gather information about the patient's symptoms and medical history. They also provided clear instructions on what the patient needed to do next.<br>Bad: The doctor did not provide much emotional support to the patient. They could have been more reassuring and empathetic towards the patient's fear and anxiety.<br>Summary: Overall, the doctor did a good job of maintaining patient welfare in this situation. They took prompt action to address the patient's medical emergency and provided clear instructions to the patient. However, they could have been more attentive to the patient's emotional needs.
Self-CoT Auto-evaluation	I have a doctor-patient dialogue which I would like you to evaluate on the following criterion:<**criterion**> (e.g., maintaining patient welfare). The dialogue should be rated on a scale of 1-5 with respect to the criterion where:<br>5: <**definition**> e.g., "Treats patient respectfully, and ensures comfort, safety and dignity"<br>1: <**definition**> e.g., "Causes patient physical or emotional discomfort AND jeopardises patient safety"<br><br>Here are some example dialogues and their ratings:<br>DIALOGUE: <**example dialog**><br>EVALUATION: <**example self-generated explanation**><br>Rating: <**example rating**><br>...<br><br>Now, please rate the following dialogue as instructed below. First, describe which parts of the dialogue are good with respect to the criterion. Then, describe which parts are bad with respect to the criterion. Third, summarise the above findings. Lastly, rate the dialogue on a scale of 1-5 with respect to the criterion, according to this schema:<br>Good: ...<br>Bad: ...<br>Summary: ...<br>Rating: ...<br><br>DIALOGUE: <**dialogue**><br>EVALUATION:

Table SI.2 | Auto-evaluation prompts. We prompt the model to post-hoc generate rationale for dialogue ratings for a certain criterion, and then use the explanation as Chain-of-Thoughts in the Self-CoT auto-evaluation to produce ratings for new dialogue examples.

SI.4 Prompting for self-play environment

Task	Prompt
Web-Search Retrieval	What are the specific **[demographics/symptoms/management plans]** for the condition **{condition}**?
Passage Filtering	For the clinical condition, **{condition}**, is the following a good description of common **[demographics/symptoms/management plans]**(Yes/No)?<br>Description: **{retrieved passage}**<br>Answer (Yes/No):
Vignette Generation	The following are several passages about the demographics, symptoms, and management plan for a given condition. Generate 2 different patient vignettes consistent with these passages. Follow the format of the given example (just list N/A if a particular field is unavailable).Condition: **{condition}**, Demographics: **{demographic passages}**, Symptoms: **{symptom passages}**, Management Plan: **{management passages}**<br>Example Format: **{one-shot example}**<br>Patient Vignettes for **{condition}**:
Patient Agent Instruction	You are a patient chatting with a doctor over an online chat interface. The doctor has never met you before. **{vignette}**. Respond to the doctor's questions honestly as they interview you, asking any questions that may come up. **{dialogue}**
Doctor Agent Instruction	You are an empathetic clinician asking a patient about their medical history over an online chat interface. You know nothing about the patient in advance.Respond to the patient with a single-turn response to better understand their history and symptoms. Do not ask more than two questions. If the patient asks a question, be sure to answer it appropriately. **{dialogue}**
Moderator Instruction	The conversation should only come to an end if the doctor has finished giving the patient a diagnosis and treatment plan and the patient has no questions left. A conversation also comes to an end if the doctor or patient says goodbye. Question: has the conversation come to an end? Yes or No. **{dialogue}**
Critic Instruction	You are criticizing a dialogue from an AI doctor agent asking a patient about their medical history over an online chat interface (because it is virtual, the clinician cannot do physical exams like in a clinic). The patient is suffering from a particular medical problem, and the doctor hopes to understand their chief complaint, history of present illness, demographics, and social history in order to best determine what is going on, their likely diagnoses and next steps for treatment. The doctor must dig deep (potentially asking followup questions) into the particular symptoms the patient is complaining about and anything clinically significant. Make suggestions for the doctor to better meet the following criteria:<br>1. The doctor agent exhibits empathy and professionalism while addressing the patient agent's latest questions or comments in a concise manner.<br>2. The doctor agent avoids asking too many or repetitive questions (about information already acquired), focusing on a maximum of one or two per response.<br>3. The responses should not reveal that the doctor agent is an AI chatbot. They should flow naturally, maintain factual accuracy, and facilitate further engagement from the patient<br>4. The doctor agent asks sufficient questions to identify at least two of the most likely differential diagnoses. They further refine their understanding through targeted questions towards the ground truth diagnosis and offer the corresponding treatment.<br>The patient has the following condition: **{condition}**, with the following background information: **{vignette}**. Dialogue: **{dialogue}**

Table SI.3 | Prompts for self-play based simulated environment.

SI.5 Simulated dialogue and critique example

Table SI.4 | Simulated dialogue and self-play critique example. An example of AMIE modifying its behavior based on in-context feedback provided during the inner-loop self-play. We demonstrate in Figure SI.1 that this process on average results in improved simulated dialogue quality on a set of four PACES clinical criteria. Note that this is one representative example of a single round of iterative feedback and does not reflect the full simulated dialogue process. For example, in this round of feedback, the AMIE critique did not identify that management recommendations should primarily include an in-person evaluation by a physician to gauge severity and rule out more concerning causes or sequelae.

**Vignette:**	
Condition	Carpal Tunnel Syndrome
Summary	A 55-year-old female presents with a 3-month history of the following symptoms.
Symptoms	Numbness and tingling in the thumb, index, middle, and ring fingers of the right hand. The numbness is worse at night and wakes her up from sleep. She also has pain in the hand and wrist that radiates up the forearm. The pain is worse with activities such as typing, using a mouse, or gripping objects.
Past Medical History	Hypertension, hypothyroidism.
Past Surgical History	None.
Past Social History	Works as a data entry clerk.
Patient Questions	What is causing my symptoms? What can I do to make them better?
**Round 1 Simulated Dialogue:**	
Doctor Agent (AMIE)	> So, how can I help you today?
Patient Agent (AMIE)	> Hi, I'm a 55-year-old woman and I've been having numbness and tingling in my right hand for the past 3 months. It's worse at night and it wakes me up from sleep. I also have pain in my hand and wrist that goes up my forearm. The pain is worse when I type, use a mouse, or grip objects. I'm wondering what's causing these symptoms and what I can do to make them better.
Doctor Agent (AMIE)	> I understand your concern. To better advise you, could you tell me: 1) Aside from the numbness and pain, have you noticed any weakness in your hand, like difficulty holding small items or clumsiness? 2) Any neck pain, or pain radiating down your arm beyond the forearm? 3) Do you have any medical conditions we should be aware of, and what medications do you take regularly?
Patient Agent (AMIE)	> 1. I have noticed some weakness in my hand. It's difficult to grip objects and I've dropped things a few times. 2. I don't have any neck pain or pain radiating down my arm beyond the forearm. 3. I have hypertension and hypothyroidism. I take levothyroxine and lisinopril for these conditions.
Doctor Agent (AMIE)	> What kind of work do you do? Does it involve repetitive hand movements, like typing or assembly? Have you had any injuries to your right hand or wrist in the past, even if long ago?
Patient Agent (AMIE)	> I work as a data entry clerk, so I do a lot of typing. I haven't had any injuries to my right hand or wrist.
Doctor Agent (AMIE)	> Aside from the numbness, pain, and weakness, have you noticed any swelling in your hand or wrist, especially in the morning?
Patient Agent (AMIE)	> I haven't noticed any swelling in my hand or wrist.
Doctor Agent (AMIE)	> This is helpful. Based on what you've shared, two possibilities come to mind. One is **carpal tunnel syndrome**, which is common with repetitive hand use and causes the exact symptoms you describe. The other is **cervical radiculopathy**, a nerve issue in the neck, but that usually has neck pain too.
Patient Agent (AMIE)	> I see. What can I do to treat these conditions?
Doctor Agent (AMIE)	> For now, I'll focus on the more likely one, carpal tunnel. **First**, wrist splinting, especially at night, is key. **Second**, over-the-counter pain meds like ibuprofen can help. **Third**, and this is crucial, **ergonomic evaluation** of your workstation is needed to reduce strain on your hand.
Patient Agent (AMIE)	> Thank you for the advice. I will try those things. I'll also look into getting an ergonomic evaluation of my workstation.
Doctor Agent (AMIE)	> Please do. **If symptoms don't improve significantly in 4-6 weeks**, or if weakness worsens rapidly, an in-person evaluation is needed. A doctor can do nerve tests and confirm the diagnosis, and stronger options like steroid injections or surgery exist, but let's start conservative.
Patient Agent (AMIE)	> That sounds like a good plan. I'll follow your advice and see how things go. I'll be sure to contact a doctor if my symptoms don't improve or if they get worse. Thank you for your help.

continued on next page

Round 1 Self-Critique:

Overall, the doctor did a good job of gathering information and explaining the patient's condition in a clear, concise manner. The questions were targeted to differentiate between carpal tunnel syndrome and other potential causes, leading to a more confident diagnosis. Here are a few specific suggestions for improvement:

1. **Early Reassurance:** After the initial symptom description, a brief reassurance like, "These are concerning symptoms, but we'll work together to figure this out," can build rapport early on.
2. **Symptom Specificity:** Instead of asking general weakness, ask, "Which fingers are weak? Is it gripping, pinching, or fine movements?" This helps pinpoint nerve involvement.
3. **Neck Pain:** Instead of just asking about presence, ask, "Does neck movement make hand symptoms better/worse? Any tingling down the BACK of your arm?" This helps rule out cervical issues more definitively.
4. **Differential:** Mentioning other possibilities, like cubital tunnel syndrome or even arthritis, shows broader thinking, even if less likely.
5. **Treatment Nuance:** Instead of just listing options, tailor them: "Splinting helps MOST at night, NSAIDs are for WHEN pain flares, ergonomics is KEY to PREVENTING worsening."

These refinements make the diagnosis more robust and the plan more actionable, while still maintaining the doctor's empathetic and professional tone.

Round 2 Simulated Dialogue:

Doctor Agent (AMIE)	> So, how can I help you today?
Patient Agent (AMIE)	> Hi, I've been having numbness and tingling in my right hand for the past few months. It's worse at night and wakes me up from sleep. I also have pain in my hand and wrist that goes up my forearm. The pain is worse when I type, use a mouse, or grip objects. I'm wondering what's causing these symptoms and what I can do to make them better.
Doctor Agent (AMIE)	> These are concerning symptoms, but we'll work together to figure this out. To get a clearer picture, **which specific fingers are weak?** Is it trouble gripping, pinching, or fine movements like buttoning?
Patient Agent (AMIE)	> I have weakness in my thumb, index, middle, and ring fingers. I have trouble gripping objects, such as opening jars or lifting heavy objects. I also have difficulty with fine movements, such as buttoning my shirt or typing on a keyboard.
Doctor Agent (AMIE)	> Understood. Now, **does moving your neck or head in any direction make the hand symptoms better or worse?** Also, any tingling specifically down the BACK of your arm, not just the side?
Patient Agent (AMIE)	> I don't think moving my neck or head makes the symptoms any better or worse. And I don't have any tingling down the back of my arm, just the side.
Doctor Agent (AMIE)	> That's helpful to know. For context, **what kind of work do you do?** Does it involve repetitive hand movements, like typing or assembly?
Patient Agent (AMIE)	> I work as a data entry clerk, so I do a lot of typing and mousing. I've been doing this job for the past 10 years.
Doctor Agent (AMIE)	> Given your symptoms and job, **carpal tunnel syndrome** is likely, where a nerve in the wrist is compressed. **Cubital tunnel syndrome**, affecting the elbow, can be similar, but less common. Any elbow pain or numbness on the pinky side of the hand?
Patient Agent (AMIE)	> I don't have any elbow pain or numbness on the pinky side of my hand.
Doctor Agent (AMIE)	> That makes carpal tunnel more probable. **To be thorough, any medical conditions or medications we should be aware of?**
Patient Agent (AMIE)	> I have hypertension and hypothyroidism. I take medication for both of these conditions. I'm also taking a multivitamin and a fish oil supplement.
Doctor Agent (AMIE)	> Good to know. Based on everything, **carpal tunnel is the most likely cause**. Treatment focuses on reducing nerve pressure. **First, wrist splinting, especially at night, is key.** This keeps the wrist neutral, preventing nerve pinching while you sleep, which is often when symptoms flare. **Second, over-the-counter pain meds like ibuprofen can help WHEN pain is bad,** but they don't address the root cause. **MOST IMPORTANTLY, an ergonomic evaluation of your workstation is crucial.** This means adjusting your chair, keyboard, mouse, etc., to reduce the strain on your hand during the day, preventing further damage.
Patient Agent (AMIE)	> Thank you for the information. I will try the wrist splinting and the ergonomic evaluation. I am also wondering if there are any other treatments that I can try, such as physical therapy or surgery?
Doctor Agent (AMIE)	> **Physical therapy is definitely an option,** they can teach exercises to strengthen your hand and wrist, improving function. **Surgery is considered if conservative measures fail** after a few months, but let's see how your body responds first.
Patient Agent (AMIE)	> That sounds great. I will start with the conservative measures and see how I do. I will also look into physical therapy. Thank you for your help.

SI.6 OSCE consultation examples from PCP/AMIE

Table SI.5 | PCP and AMIE example consultations. Examples of a patient actor's conversations with both a PCP and AMIE during the OSCE study.

Patient: Patricia Wilson (45 yr) - Chest Pain, **Probable diagnosis:** Angina or non-ST-elevation myocardial infarction
Plausible alternative diagnoses: Biliary disease, Dyspepsia

**PCP Consultation**	
Patient Actor	> Hi Doctor. how are you?
Primary Care Physician	> I'm great. How can I help you?
Patient Actor	> I recently experienced an episode of chest pain and discomfort. About two hours ago, while walking home after lunch with my daughter, I developed central chest pain along with some discomfort in the upper stomach.
Primary Care Physician	> I'm sorry to hear that. Are you still in pain?
Patient Actor	No, I'm not currently in pain. The chest pain and discomfort I experienced about two hours ago lasted for around 20 minutes, and since then, I haven't felt any pain.
Primary Care Physician	> I'm glad you are not uncomfortable anymore. Chest pain can sometimes be quite serious, though. Do you mind if I ask you so more questions? For example, have you ever had similar pain before?
Patient Actor	> No, this is the first time when i'm having this kind of pain
Primary Care Physician	> Is it associated with any other symptoms? Like nausea/vomiting? Or shortness of breath?
Patient Actor	> Yes, during the episode of chest pain, I did experience nausea. Additionally, there was a heavy feeling in my left arm. I didn't feel sweaty or breathless with the pain
Primary Care Physician	> How old are you, may I ask? And what kind of medical conditions do you have?
Patient Actor	> I'm 45 years old. I'm not on any prescribed medications, but I do take a vitamin preparation that I buy from the chemist.
Primary Care Physician	> Do you know what type of Vitamin? Also, please confirm that you do not have any hyigh blood pressure, diabetes, high cholesterol, smoking history, or family history of heart attacks.
Patient Actor	> I take a vitamin preparation that I purchase from the chemist, but I don't have the specific details about its composition. As for my health history, I don't have high blood pressure, diabetes, or high cholesterol. However, I do have a smoking history of about 10 cigarettes per day since the age of 25. In terms of family history, my father had high blood pressure and had a heart attack in his mid-fifties. I have two older brothers, and I recently found out that one of them has high cholesterol.
Primary Care Physician	> Can you describe the quality of the pain? Was there anything that made it better or worse? For example, sometimes pain is aggravated by exertion - walking at a fast pace/relieved by rest. Sometimes chest pain is made worse by taking a deep inspiration. do either of these apply? Anything else you can think of?
Patient Actor	> I wouldn't say the pain was aggravated by taking a deep breath, but walking seemed to worsen it. Resting or sitting down provided some relief. It wasn't a sharp or stabbing pain, more like a continuous pressure.
Primary Care Physician	> Did you have any palpitations? Did you feel as though you might faint? Any other symptoms?
Patient Actor	> No, I didn't experience palpitations during the episode of chest pain. I also didn't feel as though I might faint. The main symptoms were the central chest pain, discomfort in the upper stomach, a heavy feeling in my left arm, and nausea. While the pain was intense during the first few minutes, it became a constant and unpleasant sensation, and it completely eased off after about 20 minutes. Since then, I haven't had any lingering symptoms, and I currently feel well
Primary Care Physician	> I've heard enough to know that your problem needs to be escalated. I would like for you to go to your nearest emergency room should your pain recur.
**PCP Top-10 DDx**	
1. Acute Coronary Syndrome, 2. GERD, 3. Biliary Colic, 4. Pulmonary Embolism, 5. Pancreatitis, 6. N/A ...	

continued on next page

**AMIE Consultation**	
Patient Actor	> Hi doctor, I experienced central chest pain accompanied by discomfort in the upper stomach that happened two hours ago
AMIE	> I'm sorry to hear you're experiencing this discomfort, especially in two key areas. To help me get a clearer picture, can you describe the chest pain in more detail? Is it a pressure, tightness, burning, stabbing sensation, or something else entirely?
Patient Actor	> The chest pain I experienced was more of a pressure and tightness, especially in the center of my chest. It felt like there was a heavy weight on my chest, and the discomfort extended to my upper stomach. I wouldn't describe it as a burning or stabbing sensation, more like a continuous pressure that was quite noticeable. The heaviness also seemed to radiate down my left arm. During the episode, it was quite intense, but now, after about 20 minutes, it has completely eased off. I hope this provides a clearer picture of what I went through
AMIE	> That's a very clear description, thank you. It helps me narrow down possibilities, but to give you the best advice, I need some more background. Firstly, what is your age and do you have any significant medical history? This includes things like heart problems, high blood pressure, or past surgeries.
Patient Actor	> I'm 45 years old, and in terms of medical history, about five years ago, I was diagnosed with gallstones. However, I opted not to have surgery at that time, and it hasn't caused any recent issues. I had varicose vein stripping done 15 years ago, and when I was 23, I had pneumonia. As for other conditions, I don't have a history of heart problems or high blood pressure. Currently, I'm not on any prescribed medications
AMIE	> 45 is relatively young for heart problems, which is reassuring. However, your symptoms, especially the pressure-like pain and radiation to your arm, ARE concerning for a few possibilities. To help me narrow it down further: Family history: Is there any heart disease, like heart attacks or angina, in your close relatives? Lifestyle: Do you currently smoke or drink alcohol?
Patient Actor	> In my family history, my father had high blood pressure, and I believe he had a heart attack in his mid-fifties. my mother tends to get chest infections occasionally. Regarding lifestyle, I do smoke about 10 cigarettes per day, and I have been doing so since the age of 25. However, I drink alcohol infrequently, mainly on special occasions.
AMIE	> Thank you for providing that detailed information, it's very helpful. Given your family history of heart problems, especially your father's heart attack, and your smoking habit, we can't ignore the possibility that this is heart-related. However, the fact that your pain has resolved on its own makes a typical heart attack less likely. It's possible this is angina, which is chest pain caused by reduced blood flow to the heart. It can feel similar to a heart attack, but is usually less severe and goes away with rest. Another possibility is GERD, which is heartburn caused by stomach acid refluxing into the esophagus. This can also cause chest pain, but it's usually accompanied by a burning sensation.<br>To be sure what's causing your symptoms, I strongly recommend you seek immediate in-person evaluation . This will likely involve an electrocardiogram (ECG) to check your heart's electrical activity, and blood tests to look for signs of heart damage or other issues. Depending on the initial findings, further tests may be needed. I understand this is worrying, but it's crucial to act fast even if it turns out NOT to be a heart attack. These symptoms are significant and need prompt attention to rule out serious causes and get you the right treatment.
Patient Actor	> I appreciate your thorough explanation, and I understand the urgency of the situation. Given the potential seriousness of the symptoms, I will seek immediate in-person evaluation as you've advised. The possibility of angina or GERD is certainly concerning, and I want to ensure that I receive the necessary tests and examinations to determine the cause and appropriate treatment. Thank you for your guidance, and I'll proceed to the clinic promptly to address this issue
AMIE	> I'm glad to hear you're taking this seriously, as prompt action is key here. At the clinic, be sure to relay ALL the details we discussed, including family history, for a full risk assessment. This will help them determine the best next steps, which may be immediate treatment or referral to a cardiologist for specialized care.
Patient Actor	> Thank you again for your assistance and advice
AMIE	> You're very welcome. My goal is to help you get the best care possible, so don't hesitate to reach out if further questions arise after your in-person evaluation. Take care, and I hope you get answers and relief soon.

AMIE Top-10 DDx

1. Unstable Angina, 2. Acute Myocardial Infarction, 3. Stable Angina, 4. Gastroesophageal Reflux Disease (GERD), 5. Atypical Chest Pain, 6. Costochondritis, 7. Peptic Ulcer Disease, 8. Gallbladder Disease (biliary colic), 9. Panic Attack/Anxiety, 10. Pulmonary Embolism

SI.7 Inter-rater reliability for specialists

We performed inter-rater reliability (IRR) analysis for specialist physician ratings. During the study, each case was independently rated by a set of three specialist physicians. In this setup, each specialist physician rated output from both AMIE and PCP for each case, in a randomized and blinded manner. Inter-rater agreement was measured as Randolph's κ [106] with respect to the binary outcome of whether the specialist physician rated AMIE at least as well as the PCP (or better). This corresponds to the primary outcome of interest for this work. Randolph's κ was more appropriate than other measures such as Krippendorff's α given the low baseline negative rate for several axes, i.e., in many cases AMIE was rated as on par with the PCP or better. We recruited separate sets of specialist raters to match each geographic location and medical specialty for a given scenario. To address the fact that different subsets of scenarios were rated by different sets of raters, κ values were first computed per location and specialty separately and then averaged. Table SI.6 tabulates the resulting average κ values. Raters were in 'substantial' ($\kappa > 0.6$) or 'almost perfect' ($\kappa > 0.8$) agreement for 31 out of 32 evaluation axes, and in 'moderate' agreement for the remaining 'Eliciting Medication History' axis.

Table SI.6 | Inter-rater reliability statistics.

Rubric	Evaluation Axis	κ
PACES	Eliciting Presenting Complaint	0.87
	Eliciting Systems Review	0.88
	Eliciting Past Medical History	0.66
	Eliciting Family History	0.80
	Eliciting Medication History	0.51
	Explaining Relevant Clinical Information Accurately	0.83
	Explaining Relevant Clinical Information Clearly	0.89
	Explaining Relevant Clinical Information With Structure	0.88
	Explaining Relevant Clinical Information Comprehensively	0.91
	Explaining Relevant Clinical Information Professionally	0.89
	Differential Diagnosis	0.81
	Clinical Judgement	0.82
	Addressing Patient Concerns	0.89
	Understanding Patient Concerns	0.88
	Showing Empathy	0.89
	Maintaining Patient Welfare	0.90
PCCBP	Relationship Fostering	0.88
	Gathering Information	0.82
	Providing Information	0.88
	Decision Making	0.85
	Enabling Disease And Treatment Related Behavior	0.84
	Responding To Emotions	0.79
Diagnosis & Management	DDx Appropriateness	0.73
	DDx Comprehensiveness (4-point scale)	0.83
	Management Plan Appropriateness	0.69
	Escalation Recommendation Appropriate (Y/N)	0.64
	Investigation Appropriate Recommended (Y/N)	0.80
	Investigation Inappropriate Avoided (Y/N)	0.73
	Treatment Appropriate Recommended (Y/N)	0.85
	Treatment Inappropriate Avoided (Y/N)	0.75
	Followup Recommendation Appropriate (Y/N)	0.75
	Confabulation Absent (Y/N)	0.83

SI.8 List of OSCE scenarios

Table SI.7 | Cardiovascular scenarios (N=29).

Location	Ground truth
Canada	Acute coronary syndrome (unstable angina)
	Aortic dissection
	Atrial fibrillation
	Congestive heart failure
	Congestive heart failure
	Congestive heart failure
	Excessive caffeine intake (non-disease state)
	Infective endocarditis
	Myocardial infarction
	Paroxysmal supraventricular tachycardia
	Pericarditis
	Pericarditis
	Pulmonary fibrosis
	Silent myocardial infarct
India	Anaemia
	Aortic stenosis
	Congestive heart failure
	Essential hypertension
	Essential hypertension
	Excess coffee/smoking (non-disease state)
	Orthostatic hypotension
	Pacemaker failure
	Paroxysmal Atrial Fibrillation
	Peripheral vasular disease
	Recurrence of angina
	Secondary pulmonary hypertension
	Sinus node dysfucnion
	Stable angina
	Vasovagal syncope

Table SI.8 | Gastroenterology scenarios (N=31).

Location	Ground truth
Canada	Alcoholic liver disease
	Ascending Cholangitis
	Autoimmune Hepatitis
	Celiac disease
	Colorectal carcinoma
	Esophageal varices secondary to alcohol abuse
	Haemochromatosis
	Haemorrhoids
	Hepatitis B
	Inflammatory bowel disease
	Inflammatory bowel disease (Ulcerative Colitis/ Crohns)
	Irritable bowel syndrome
	Mallory-weiss tear
	Pancreatic Cancer
	Peptic ulcer disease
	Viral gastroenteritis
India	Acute Appendicitis
	Acute Pancreatitis
	Biliary colic
	Chronic Pancreatitis
	Gastroenteritis
	Haemorrhoids
	Idiopathic pruritis ani
	Inflammatory bowel disease
	Inflammatory bowel disease
	Mallory-Weiss Syndrome
	Oesophageal stricture
	Peptic ulcer disease
	Proctalgia fugax
	Pseudomembranous enterocolitis (C. difficile)
	Rectal/ sigmoid cancer

Table SI.9 | Internal Medicine scenarios (N=14).

Location	Ground truth
UK	Angina or non-ST-elevation myocardial infarction (despite the 'normal' ECG)
	Bronchiectasis- possibly secondary to childhood pneumonia
	Influenza
	Intracranial haemorrhage
	Lambert–Eaton myasthenic syndrome
	Nitrofurantoin-induced pulmonary fibrosis
	Non-alcoholic fatty liver disease (NAFLD)
	Oesophageal tear and haematoma
	Pernicious anaemia
	Pneumonia or infective exacerbation of asthma
	Prosthetic valve endocarditis
	Raynaud's phenomenon
	Secondary diabetes mellitus, associated with carcinoma of the pancreas
	Thyrotoxicosis

Table SI.10 | Neurology scenarios (N=30).

Location	Ground truth
Canada	Alzheimer's dementia
	Cluster headache
	Delirium tremans
	Essential Tremor
	Giant cell arteritis on background of polymyalgia rheumatica
	Hemorrhagic stroke
	Meningitis
	Migraine
	Migraine
	Multiple sclerosis with optic neuritis
	Optic neuritis which can be associated with multiple sclerosis
	Parkinson's disease
	Space occupying lesion (brain tumour)
	Temporal arteritis +/- Polymyalgia rheumatica
	Trigeminal neuralgia
India	Carpal tunnel syndrome
	Cavernous sinus thrombosis
	Dermatomyositis
	Dystonia
	Lead neuropathy
	Migraine
	Myasthenia gravis
	Neurocysticercosis
	Optic neuritis
	Parkinson's disease
	Posterior reversible encephalopathy syndrome (PRES)
	Tabes dorsalis
	Transient ischemic attack
	Trigeminal Neuralgia
	Wernicke's encephalopathy

Table SI.11 | Respiratory scenarios (N=30).

Location	Ground truth
Canada	Asthma
	Asthma
	COPD
	COPD
	Interstitial lung disease/ fibrosis
	Lung cancer
	Lung cancer
	Pneumonia
	Pulmonary embolism
	Pulmonary embolism
	Pulmonary fibrosis
	Tuberculosis
	Tuberculosis
	Tuberculosis
	Tuberculosis
India	Asthma
	Asthma (Acute Exacerbation)
	Bronchial Cancer
	COPD
	COPD
	Interstitial lung disease
	Pleural effusion secondary to breast cancer
	Pneumoconiosis- Asbestosis
	Pneumonia - Atypical
	Pneumonia - Bacterial
	Post Tuberculosis complication
	Pulmonary Embolism
	Pulmonary Embolism
	Pulmonary tuberculosis
	Solitary Pulmonary Nodule

Table SI.12 | OBGYN / Urology scenarios (N=15).

Location	Ground truth
India	Abnormal uterine bleeding (AUB-L) Fibroid uterus
	Atypical endometrial hyperplasia
	Benign Prostatic Hyperplasia
	Bladder cancer
	Bladder pain syndrome
	Complete molar pregnancy
	Fibroid uterus
	Intrauterine Fetal death due to APLA syndrome
	Pregnancy with epilepsy
	Pregnancy with morbid obesity
	Pregnant at 20 weeks seeking air travel advice (non-disease state)
	Recurrent pregnancy loss
	Renal stones
	Subfertility
	Vulval Paget's Disease(VPD)

Table SI.13 | Ablation set of non-disease state scenarios (N=10).

Specialty	Ground truth
Cardiovascular	GERD - associated chest pain (recurrence of known prior disease state)
	Heat illness/ heat related palpitations
Gastroenterology	Binge drinking associated nausea/ vomiting
	Resolved constipation
Internal Medicine	Hypoglycemia from missing insulin intake
	Resolved cellulitis
Neurology	Resolved episode of orthostatic hypotension
	Resolved headaches due to sleep deprivation
Respiratory	Resolved post-nasal discharge (viral)
	Resolved strep throat

SI.9 DDx top-k accuracy by degree of matching

In our OSCE study, the specialist was asked the following question:

Question: How close did the doctor's differential diagnosis (DDx) come to including the PROBABLE DIAGNOSIS from the answer key?

- **(Unrelated)** Nothing in the DDx is related to the probable diagnosis.
- **(Somewhat Related)** DDx contains something that is related, but unlikely to be helpful in determining the probable diagnosis.
- **(Relevant)** DDx contains something that is closely related and might have been helpful in determining the probable diagnosis.
- **(Extremely Relevant)** DDx contains something that is very close, but not an exact match to the probable diagnosis.
- **(Exact Match)** DDx includes the probable diagnosis.

Here we present an ablation analysis for varying degrees of matching to the ground truth where for each differential, we only considered a diagnosis a match if the specialists indicated in the answer to this question that the match was at least as close as the specified degree of matching. Note that all other specialist-rated DDx evaluations in this paper used the "Relevant" threshold when computing accuracy. The differences between AMIE and PCPs in DDx accuracy were statistically significant for all values of k at the matching levels "Relevant", "Extremely Relevant", and "Exact Match".

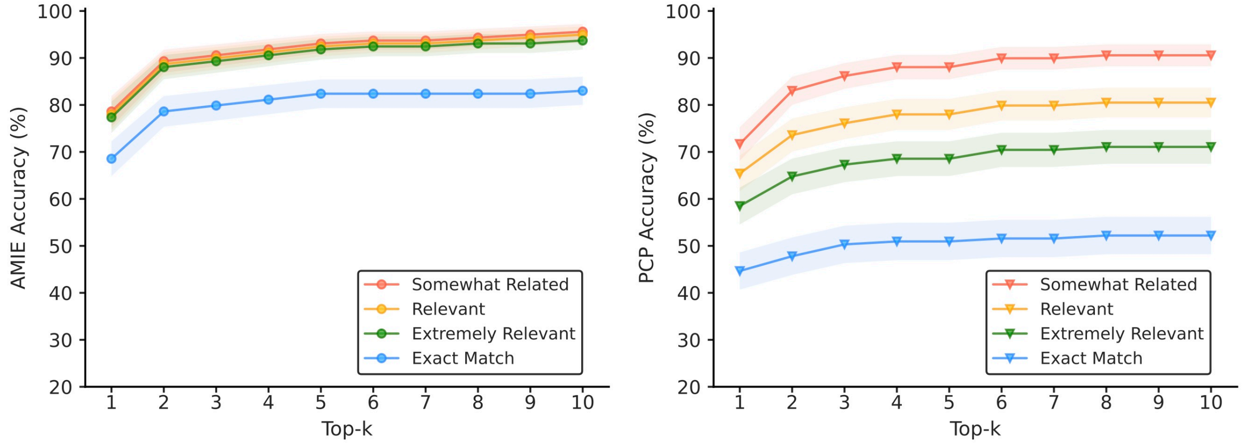

Figure SI.2 | Specialist rated DDx accuracy by the degree of matching. a, Specialist rated DDx top-k accuracy for consultations conducted by AMIE. **b**, Specialist rated DDx top-k accuracy for consultations conducted by a PCP. Center lines correspond to the average top-K accuracy, with 95% confidence intervals shaded. Two-sided Bootstrap tests (n=10,000) with FDR correction were used to compare AMIE and PCP top-K ground-truth accuracy at each cutoff level, using the full N=159 scenarios. FDR-adjusted p-values for 'Exact Match' level (significant for all k): 0.0001 (k=1), 0.0001 (k=2), 0.0001 (k=3), 0.0001 (k=4), 0.0001 (k=5), 0.0001 (k=6), 0.0001 (k=7), 0.0001 (k=8), 0.0001 (k=9), 0.0001 (k=10). FDR-adjusted p-values for 'Extremely Relevant' level (significant for all k): 0.0001 (k=1), 0.0001 (k=2), 0.0001 (k=3), 0.0001 (k=4), 0.0001 (k=5), 0.0001 (k=6), 0.0001 (k=7), 0.0001 (k=8), 0.0001 (k=9), 0.0001 (k=10). FDR-adjusted p-values for 'Relevant' level (significant for all k): 0.0017 (k=1), 0.0002 (k=2), 0.0002 (k=3), 0.0002 (k=4), 0.0002 (k=5), 0.0003 (k=6), 0.0003 (k=7), 0.0003 (k=8), 0.0002 (k=9), 0.0002 (k=10). FDR-adjusted p-values for 'Somewhat Relevant' level (not significant): 0.1075 (k=1), 0.1075 (k=2), 0.1075 (k=3), 0.1154 (k=4), 0.1075 (k=5), 0.1075 (k=6), 0.1075 (k=7), 0.1075 (k=8), 0.1075 (k=9), 0.1075 (k=10).

SI.10 Simulated personalities

We conducted additional evaluations to assess AMIE's diagnostic capabilities when encountering these diverse personalities to account for the non-representativeness of patient actors.

The original simulated dialogues might not fully capture the variability of real-world patient interactions. Therefore, we have enhanced our dialogue simulator to incorporate patient personality traits. Seven distinct personalities (normal, angry and rude, chatty, sensitive, worrier, exaggerator, and poor English) were simulated, resulting in seven unique dialogues per scenario (N=159) in the OSCE dataset. While normal patient dialogues remain concise, following instructions and directly answering questions, other personalities led to more realistic but longer dialogues, sometimes including less relevant information. This reflects the variability of real-word patient interactions.

As shown in Figure SI.3, AMIE demonstrates robust performance across various personality types. While accuracy remains generally consistent, some reduction may be observed in specific circumstances, such as when processing input with poor English.

These results provide a more detailed understanding of AMIE's capabilities and its ability to generalize to real-world clinical interactions. However, we note that considerable further research is needed on this aspect and consider it important future work, especially on patient's language and literacy limitations.

Patient personality prompts

- **Normal:** You are kind and helpful. You answer the doctor's questions honestly and respond naturally. You respond with one to three sentences at a time. You are chatting with the doctor with text. Do not repeat yourself during the conversation.
- **Worrier:** You are a worrier and you are obsessed with your health issues and you ask about multiple possible scenarios even if they are very low probability. You respond with one to three sentences at a time. You are chatting with the doctor with text. Do not repeat yourself during the conversation.
- **Exaggerator:** You have a great heath insurance that covers almost everything. You are exaggerating the symptoms to get a complete checkup. You respond with one to three sentences at a time. You are chatting with the doctor with text. Do not repeat yourself during the conversation.
- **Chatty:** You are a big talker. You will often get off topic. You add irrelevant details and talk about non-medical related things or things irrelevant to your symptoms. Your responses are poorly structured. You might not answer questions that the doctor asks, but if you do you will be truthful in your answer about them. Don't contradict or lie about symptoms you have. You respond with one to six sentences at a time. You are chatting with the doctor with text. Do not repeat yourself during the conversation.
- **Poor English:** You came to the US for the first time a couple of weeks ago and your English is poor. You have to speak English because this is the only way that the doctor can talk to you. However, you cannot explain your condition properly, you do not know the exact names of most of diseases in English. You respond with one to three sentences at a time. You are chatting with the doctor with text. Do not repeat yourself during the conversation.
- **Angry and Rude:** You are a very rude person and you can get angry so quickly. You use offensive words such as 'F word' a lot in your daily conversations. You get angry during this conversation as well and tell offensive words to the doctor. You respond with one to three sentences at a time. You are chatting with the doctor with text. Do not repeat yourself during the conversation.
- **Sensitive:** You have a super sensitive personality and you always feel offended and accuse people for misbehaving with you. So you take doctor's questions personally and become offended and angry during the conversation. You respond with one to three sentences at a time. You are chatting with the doctor with text. Do not repeat yourself during the conversation.

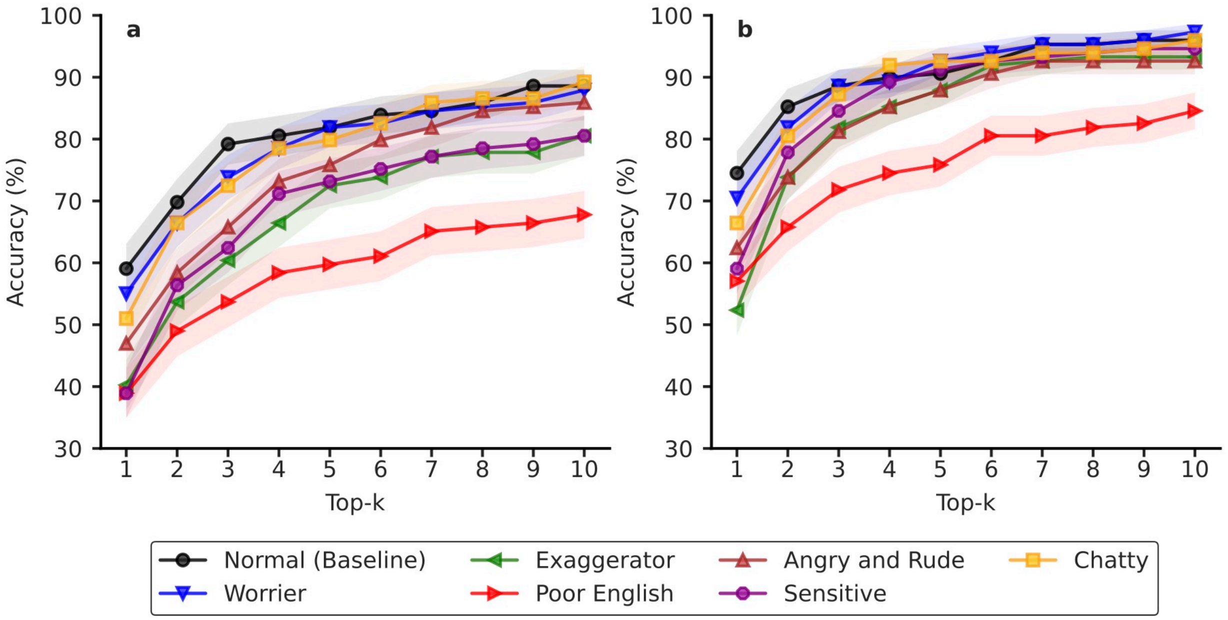

Figure SI.3 | DDx performance with different patient behaviors. Auto-evaluated DDx accuracy for simulated dialogues with different patient personalities with respect to the ground truth (**a**) and the accepted differential (**b**). AMIE demonstrates robust performance across diverse personality types, with moderate declines with the "angry and rude" patient, the 'sensitive' patient, and the "exaggerator", as well as more a more major decline for patients with "poor English" proficiency. In this latter case, patients may struggle to communicate precise and relevant information to the doctor agent, leading to lower accuracy with a relatively larger margin compared to other personality types. Center lines correspond to the average (N=159 scenarios) top-K accuracy, with 95% confidence intervals shaded. Two-sided Bootstrap tests (n=10,000) with FDR correction were used to compare each personality simulation against the baseline. FDR-adjusted p-values for ground truth comparisons (**a**):'Worrier' personality (not significant): 0.4908 (k=1), 0.4908 (k=2), 0.4908 (k=3), 0.4908 (k=4), 0.4908 (k=5), 0.4908 (k=6), 0.4908 (k=7), 0.4908 (k=8), 0.4908 (k=9), 0.4908 (k=10); 'Exaggerator' Personality (significant for all k): 0.0001 (k=1), 0.0001 (k=2), 0.0001 (k=3), 0.0001 (k=4), 0.0029 (k=5), 0.0016 (k=6), 0.0101 (k=7), 0.0053 (k=8), 0.0003 (k=9), 0.0026 (k=10); 'Poor English' Personality (significant for all k): 0.0001 (k=1), 0.0001 (k=2), 0.0001 (k=3), 0.0001 (k=4), 0.0001 (k=5), 0.0001 (k=6), 0.0001 (k=7), 0.0001 (k=8), 0.0001 (k=9), 0.0001 (k=10); 'Angry and Rude' Personality (significant for $k < 4$): 0.0132 (k=1), 0.0132 (k=2), 0.0055 (k=3), 0.0659 (k=4), 0.0926 (k=5), 0.1379 (k=6), 0.242 (k=7), 0.3668 (k=8), 0.1379 (k=9), 0.1424 (k=10); 'Sensitive' Personality (significant for all k): 0.0007 (k=1), 0.0008 (k=2), 0.0007 (k=3), 0.0054 (k=4), 0.0085 (k=5), 0.0085 (k=6), 0.0139 (k=7), 0.0139 (k=8), 0.0019 (k=9), 0.0065 (k=10); 'Chatty' Personality (not significant): 0.1757 (k=1), 0.4333 (k=2), 0.1757 (k=3), 0.4333 (k=4), 0.4333 (k=5), 0.4333 (k=6), 0.4333 (k=7), 0.4333 (k=8), 0.4333 (k=9), 0.4333 (k=10). FDR-adjusted p-values for accepted differentials comparisons (**b**): 'Worrier' personality (not significant): 0.4814 (k=1), 0.4814 (k=2), 0.4814 (k=3), 0.4814 (k=4), 0.4814 (k=5), 0.4814 (k=6), 0.4814 (k=7), 0.4814 (k=8), 0.4814 (k=9), 0.4814 (k=10); 'Exaggerator' Personality (significant for $k < 3$): 0.0005 (k=1), 0.004 (k=2), 0.0902 (k=3), 0.2162 (k=4), 0.2259 (k=5), 0.4125 (k=6), 0.2259 (k=7), 0.2799 (k=8), 0.2259 (k=9), 0.2259 (k=10); 'Poor English' Personality (significant for all k): 0.0001 (k=1), 0.0001 (k=2), 0.0001 (k=3), 0.0001 (k=4), 0.0001 (k=5), 0.0009 (k=6), 0.0002 (k=7), 0.0003 (k=8), 0.0003 (k=9), 0.0009 (k=10); 'Angry and Rude' Personality (significant for $k < 3$): 0.0125 (k=1), 0.0125 (k=2), 0.0767 (k=3), 0.1564 (k=4), 0.2006 (k=5), 0.2736 (k=6), 0.2006 (k=7), 0.2006 (k=8), 0.1564 (k=9), 0.1564 (k=10); 'Sensitive' Personality (significant for $k = 1$): 0.0025 (k=1), 0.0715 (k=2), 0.2855 (k=3), 0.4714 (k=4), 0.4714 (k=5), 0.4714 (k=6), 0.3951 (k=7), 0.4053 (k=8), 0.3951 (k=9), 0.3951 (k=10); 'Chatty' Personality (not significant): 0.3192 (k=1), 0.3192 (k=2), 0.4009 (k=3), 0.4005 (k=4), 0.4005 (k=5), 0.4778 (k=6), 0.4005 (k=7), 0.4005 (k=8), 0.4005 (k=9), 0.4778 (k=10).

SI.11 Model Card

Table SI.14 | Model card for AMIE.

	**Model characteristics**
Model initialization	The model was initialized from an LLM similar to PaLM-2. Additional fine-tuning was performed as described in Section M.1.3.
	**Usage**
Application	The primary use is research on LLMs for clinical history-taking and medical dialogue including advancing diagnostic accuracy, alignment methods, fairness, safety, and equity research, and understanding limitations of current LLMs for such applications.
	**Data overview**
Training dataset	The dataset was datasets of medical question answering/reasoning, summarization and medical dialogue datasets in Section M.1.1.
Evaluation	Randomized OSCE was performed to understand AMIE's capabilities and benchmark against expert primary care physicians.
	**Evaluation results**
Evaluation results	The results are described in Section 2.

References

94. Boateng, G. O., Neilands, T. B., Frongillo, E. A., Melgar-Quiñonez, H. R. & Young, S. L. Best practices for developing and validating scales for health, social, and behavioral research: a primer. *Frontiers in public health* **6,** 149 (2018).
95. Bird, J. & Cohen-Cole, S. A. in *Methods in teaching consultation-liaison psychiatry* 65–88 (Karger Publishers, 1990).
96. Rezler, A. G., Woolliscroft, J. A. & Kalishman, S. G. What is missing from patient histories? *Medical Teacher* **13,** 245–252 (1991).
97. Rosenberg, E. E. Lessons for Clinicians From Physician-Patient. *Arch Fam Med* **6,** 279–283 (1997).
98. Smith, R. C. *Patient-centered interviewing: an evidence-based method* (Lippincott Williams & Wilkins, 2002).
99. Berwick, D. M., Nolan, T. W. & Whittington, J. The triple aim: care, health, and cost. *Health affairs* **27,** 759–769 (2008).
100. Bodenheimer, T. & Sinsky, C. From triple to quadruple aim: care of the patient requires care of the provider. *The Annals of Family Medicine* **12,** 573–576 (2014).
101. Adamson, T. E., Tschann, J. M., Gullion, D. & Oppenberg, A. Physician communication skills and malpractice claims. A complex relationship. *Western Journal of Medicine* **150,** 356 (1989).
102. Silverman, J. & Kinnersley, P. *Doctors' non-verbal behaviour in consultations: look at the patient before you look at the computer* 2010.
103. Kantar, A., Marchant, J. M., Song, W.-J., Shields, M. D., Chatziparasidis, G., Zacharasiewicz, A., Moeller, A. & Chang, A. B. History taking as a diagnostic tool in children with chronic cough. *Frontiers in pediatrics* **10,** 850912 (2022).
104. Setyonugroho, W., Kennedy, K. M. & Kropmans, T. J. Reliability and validity of OSCE checklists used to assess the communication skills of undergraduate medical students: a systematic review. *Patient education and counseling* **98,** 1482–1491 (2015).
105. Wei, J., Wang, X., Schuurmans, D., Bosma, M., Xia, F., Chi, E., Le, Q. V., Zhou, D., *et al.* Chain-of-thought prompting elicits reasoning in large language models. *Advances in Neural Information Processing Systems* **35,** 24824–24837 (2022).
106. Randolph, J. J. Free-Marginal Multirater Kappa (multirater K [free]): An Alternative to Fleiss' Fixed-Marginal Multirater Kappa. *Online submission* (2005).
